# Immunization with porcine epidemic diarrhea virus harbouring Fc domain of IgG enhances antibody production in pigs

**DOI:** 10.1080/01652176.2020.1773006

**Published:** 2020-06-08

**Authors:** Jung-Eun Park, Hyun Jang, Ju-Hun Kim, Bang-Hun Hyun, Hyun-Jin Shin

**Affiliations:** a Research Institute of Veterinary Medicine, Chungnam National University, Daejeon, Republic of Korea; b College of Veterinary Medicine, Chungnam National University, Daejeon, Republic of Korea; c Komipharm Co. Ltd, Ansan, Republic of Korea; d Animal and Plant Quarantine Agency, Gimcheon, Republic of Korea

**Keywords:** Swine, porcine, porcine epidemic diarrhea, PEDV, coronavirus, vaccine, Fc

## Abstract

**Background:**

Outbreaks of porcine epidemic diarrhea virus (PEDV) infection have re-emerged and spread rapidly worldwide, resulting in significant economic losses. Vaccination is the best way to prevent PEDV infection in young piglets.

**Objective:**

To enhance the efficacy of an inactivated vaccine against PEDV, we evaluated the adjuvant properties of Fc domain of IgG.

**Methods:**

Fifteen crossbred gilts (180 ∼ 210 days old) were used. Five pigs in group 1 were intramuscularly vaccinated twice at 4 weeks and 2 weeks prior to farrowing with 10^6^ TCID_50_ of inactivated PEDV. Five pigs in group 2 were intramuscularly vaccinated twice at 4 weeks and 2 weeks prior to farrowing with 10^6^ TCID_50_ of inactivated PEDV-sFc. Five pigs in group 3 were not vaccinated and served as negative controls. Serum samples were collected at farrowing and subjected to ELISA, a serum neutralizing (SN) test, and a cytokine assay. Statistical analysis was performed by a two-tailed unpaired t-test.

**Results:**

Vero cells expressing swine IgG Fc on its surface was established. When PEDV was propagated in the cells expressing the swine Fc, PEDV virion incorporated the Fc. Immunization of pigs with inactivated PEDV harbouring Fc induced significantly higher antibody production against PEDV, comparing to the immunization with normal inactivated PEDV. In addition, we observed significantly increased IFN-γ levels in sera.

**Conclusion:**

Our results indicate that Fc molecule facilitate immune responses and PEDV harbouring Fc molecule could be a possible vaccine candidate. However, a challenge experiment would be needed to investigate the protective efficacy of PEDV harbouring Fc.

## 1. Introduction

Porcine epidemic diarrhea (PED) is a swine enteric disease caused by porcine epidemic diarrhea virus (PEDV) (Duarte et al. 1993). PED transmits by fecal-oral route and induces acute watery diarrhea, vomiting, dehydration, weight loss, and anorexia (Debouck and Pensaert 1980). PEDV can infect all ages of pigs, but the disease is most serious in neonatal piglets which show high morbidity (80 ∼ 100%) and mortality (50 ∼ 80%) (Turgeon et al. 1980; Pospischil et al. 1981).

Many studies have shown that the neutralizing antibody obtained from immunized sows play a major role in eradicating PEDV in neonatal piglets (de Arriba et al. 2002). As antibodies cannot cross through the placenta of sows, piglets solely acquire its antibodies predominantly via colostrum. During early lactation, secretory IgA, IgG, and IgM are passively transferred to the piglet via colostrum and milk (de Arriba et al. 2002). Most commercially available vaccines that are in use are traditional live attenuated or inactivated/killed vaccines (Gerdts and Zakhartchouk 2017). Despite PEDV vaccines have widely used in Asia for many years, severe PEDV outbreaks have still been reported in recent years (Tian et al. 2013; Lee and Lee 2014; Kim et al. 2015; Suzuki et al. 2015). The main reason for this result is that vaccines based on classical PEDV strains failed to control the more recent virulent PEDV strains (Park et al. 2018). In addition, the efficacy of current vaccines need to be further developed and improved through further study (Paudel et al. 2014).

Receptors for Fc portion of antibody play an important role in the activation of immune reaction for infections of virus and bacteria (Huber et al. 2001; Perez-Bercoff et al. 2003; Villinger et al. 2003). Therefore, immunization with a complex of virus antigen and Fc has the potential to be an improved inactivated vaccine.

In the present study, we developed an inactivated PEDV harbouring swine IgG Fc and assessed its efficacy as vaccine in sows by determining antibody production and cytokine secretion.

## 2. Materials and methods

### 2.1. Cells and viruses

African green monkey kidney cells (Vero, CCL-81) were maintained in minimum essential medium (MEM) supplemented with 10% fetal bovine serum, 100 IU/ml penicillin, and 100 µg/ml streptomycin. Cells were maintained at 37 °C in 5% CO_2_. All reagents for cell culture were purchased from Invitrogen (Carlsbad, CA, USA). PEDV strain SM98, a Vero cell-adapted vaccine strain was propagated in Vero cells as described previously (Hofmann and Wyler 1988).

### 2.2. Plasmid

The gene coding for Fc portion of swine IgG1b containing hinge, CH2, and CH3 domains was amplified from pig spleen cDNA using specific primers (5′- GGATCCGTGGCCGGGCCCTCGGTCTT-3′and 5′- GTTTAAACTTTACCCTGAGTCTTGGA-3′). The gene coding for transmembrane domain of swine transferrin receptor (pTR) was amplified from pig spleen cDNA using specific primers (5′-AGCGGCCGCGCCACCATGATGGATCAAGCTAGA-3′ and 5′- CGCGGATCCATCTGTTTTTGATTCTACACG-3′). Subsequently, the two amplified genes coding for pTR and Fc domain were cloned in pBGFP, which codes the enhanced green fluorescent protein gene. The resulting plasmid was designated as pBGFP-pTR-dHFc.

### 2.3. Establishment of Vero-Fc cell lines

Vero cells cultured in a six-well cell culture plate were transfected with 4 μg pBGFP-pTR-dHFc using Lipofectamine2000 (Invitrogen, Carlsbad, CA, USA) according to manufacturer`s instructions. Stable cell lines were selected by Zeocin (Thermo Fisher Scientific, Waltham, MA, USA) at a concentration of 500 µg/ml. Surviving colonies were further isolated and grown in MEM supplemented with 500 µg/ml. To detect Fc expression on cell surface, cells were fixed with 4% (v/v) formaldehyde in phosphate buffered saline (PBS) for 15 min at room temperature (RT). Unspecific binding was blocked using PBS containing 5% bovine serum albumin for 30 min at RT. Cells were then incubated with FITC conjugated anti-porcine IgG (LifeSpan Biosciences, Seattle, WA, USA) for 1 h at RT. After three washes with PBS, cells were observed under fluorescence microscope. The established stable cell line was designated as Vero-Fc.

### 2.4. Generation and characterization of PEDV harboring sFc

Vero and Vero-Fc cells were infected with SM98 at a multiplicity of infection of 1. The supernatant of infected cell cultures was harvested after 24 h post-infection and centrifuged at 1000 x g for 10 min to remove cell debris. To determine Fc incorporation on PEDV, viruses in the supernatants were precipitated using polyethylene glycol (MW 20,000). The pelleted viruses were subjected to electrophoresis through 10% SDS-PAGE gel and transferred onto a polyvinyl difluoride membrane. The membrane was probed with horseradish peroxidase conjugated with anti-swine IgG and then visualized using Supersignal West Dura Extended Duration Substrate® (Thermo Fisher Scientific, Waltham, MA, USA).

### 2.5. Immunization

For virus inactivation, viruses were incubated with 2 mM ethyleneimine (BEI) at 37 °C overnight. The remaining BEI was neutralized by addition of 20% sodium thiosulfate. Complete virus inactivation was verified by the absence of viral growth in Vero cell cultures. Experimental vaccines were prepared by mixing with 4 mg carbomer as the adjuvant in 2 ml of PBS.

A total of fifteen commercial crossbred gilts (180 ∼ 210 days old) were chosen from a conventional breeding farm. The pigs were healthy and had no known involvement in a PED outbreak nor had they been vaccinated with PEDV. All animals were confirmed negative for PEDV, transmissible gastroenteritis virus (TGE), porcine deltacoronavirus, and porcine rotavirus by virus specific primers on rectal swabs, and were determined to be free of antibodies to PEDV by enzyme-linked immunosorbent assay (ELISA). Pigs were randomly divided into three groups. Five pigs in group 1 were intramuscularly vaccinated twice at 4 weeks and 2 weeks prior to farrowing with 10^6^ TCID_50_ of inactivated PEDV. Five pigs in group 2 were intramuscularly vaccinated twice at 4 weeks and 2 weeks prior to farrowing with 10^6^ TCID_50_ of inactivated PEDV-sFc. Five pigs in group 3 were not vaccinated and served as negative controls. Serum samples were collected at farrowing and subjected to ELISA, a serum neutralizing (SN) test, and a cytokine assay. Colostrum samples were collected from all pigs same day on delivery. Collected serum and colostrum samples were stored in -20 °C until use.

All the animal experiments were performed according to the protocol approved by the Institutional Animal Care and Use Committee of Chungnam National University, Republic of Korea (ethics approval number: 201803 A-CNU-049).

### 2.6. ELISA

Antibody titers of PEDV-specific IgG in serum and colostrum from immunized pigs were determined by ELISA as described previously (Park et al. 2018). In brief, microtiter plates were coated with 100 μl of SM98 (10^5^ TCID_50_/ml) overnight at 4 °C and blocked with 5% skim milk for 1 h at RT. Diluted samples were added and kept at RT for 1 h, followed by 1 h incubation of horseradish peroxidase-conjugated goat anti-swine IgG antibodies. The enzymatic activity was detected by adding 3,3′,5,5′-tetramethylbenzidine substrate, stopped with 2 N H_2_SO_4_, and then the absorbance at 450 nm was measured on a microplate reader.

### 2.7. SN test

The SN test was performed as described previously (Park et al. 2018). Briefly, sera were inactivated at 56 °C for 30 min and serially diluted 2-fold. PEDV of 200 TCID_50_/0.1 ml was mixed with an equal volume of diluted sera or colostrum and incubated for 1 h at 37 °C. Vero cells were infected with 0.1 ml of each virus-serum mixture. After 1 h at 37 °C, cells were rinsed three times with PBS and maintained in MEM containing 5 μg/ml trypsin for 5 days at 37 °C. SN titers were expressed as the reciprocals of the highest serum dilution resulting in the inhibition of cytopathic effect.

### 2.8. Cytokines assay

Concentrations of interferon gamma (IFN-γ) in sera were determined by using ELISA kit (Cusabio Biotech, Wuhan, Hubei province, China) according to the manufacturer`s protocol. Concentration of each sample was figured out by using a linear-regression equation obtained from the provided serial standards.

### 2.9. Statistical analysis

All data were expressed as means with standard error of the mean (SEM). P values were calculated by a two-tailed unpaired t-test. P values < 0.05 were considered statistically significant.

## 3. Results

### 3.1. Generation of PEDV harboring sFc

**Figure 1.**
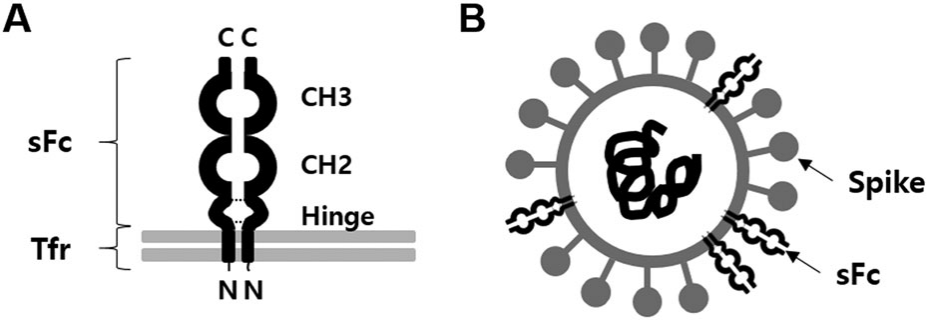
Construction of PEDV harboring sFc. (A) Scheme of the design of a reverse-Fc fusion proteins on cell surface. The chimera sFc consists with hinge, CH2, and CH3 domains and anchored by the transmembrane domain of swine transferrin (Tfr) to cellular membrane. (B) Scheme of PEDV virion harboring sFc.

We constructed genetically modified cells that express swine IgG Fc. The Fc was expressed on the cell surface, as a fusion protein (pTR-dHFc), in a reverse orientation mimicking natural IgG opsonization (Figure 1A). PEDV and several other enveloped viruses have been known to incorporate host cellular antigens with their biological functions. Therefore, PEDV grown in our genetically modified cells may acquire sFc on the viral envelope as shown in Figure 1B.

**Figure 2.**
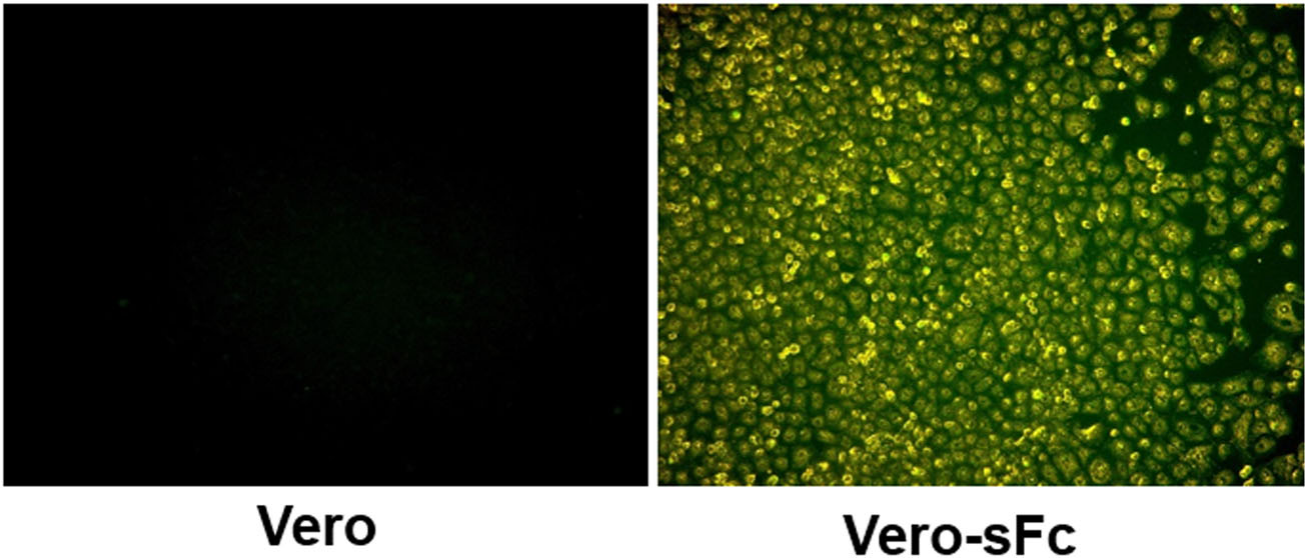
Cell surface expression of sFc. The sFc on cell surface was detected by immunocytochemistry in established zeosin resistant Vero-sFc cells and negative control Vero cells.

Vero cells were transfected with construct. Transfected cells were then selected using a zeocin marker. Selected cells were examined for the surface expression of sFc by immunofluorescence assay. As shown in Figure 2, the surface expression of sFc was observed in Vero cells containing sFc (Vero-Fc) but not in control Vero cells. The Vero-Fc cells were used for the generation of PEDV harbouring sFc.

**Figure 3.**
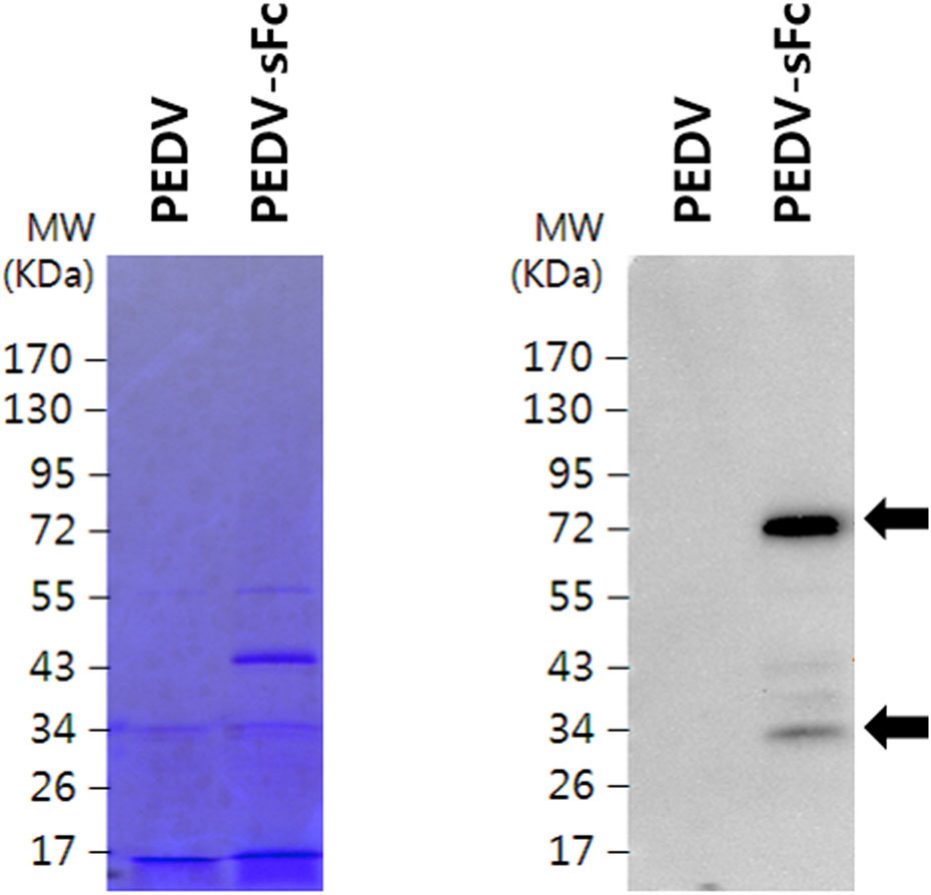
Detection of the sFc on PEDV particles. PEDV virion propagated in Vero or Vero-sFc cells were concentrated and subjected to electrophoresis in SDS-PAGE gel. Gels were stained with Coomassie blue, and sFc expression were detected by HRP-conjugated anti-swine IgG. Arrows indicate dimer and monomer forms of sFc.

We expected that PEDVs grown in Vero-Fc cells would obtain Fc fragments from the cells during virus assembly and budding. To test that, PEDVs were propagated in Vero-Fc or normal Vero cells as control. To determine sFc incorporation on PEDV particles, PEDVs were concentrated and sFc expression was determined by western blotting against swine IgG. Meanwhile, PEDV grown in normal Vero cells were used as negative control. As shown in Figure 3, concentrated PEDV-sFc, but not PEDVs grown in Vero cells, showed a distinct band at 34 kDa (a monomer form) and 72 kDa (a dimer form), corresponded to its predicted molecular weight. These results indicate that chimeric pTR-dHFc construct is able to incorporate PEDV.

### 3.2. PEDV-sFc enhances a humoral immune response in pigs

**Figure 4.**
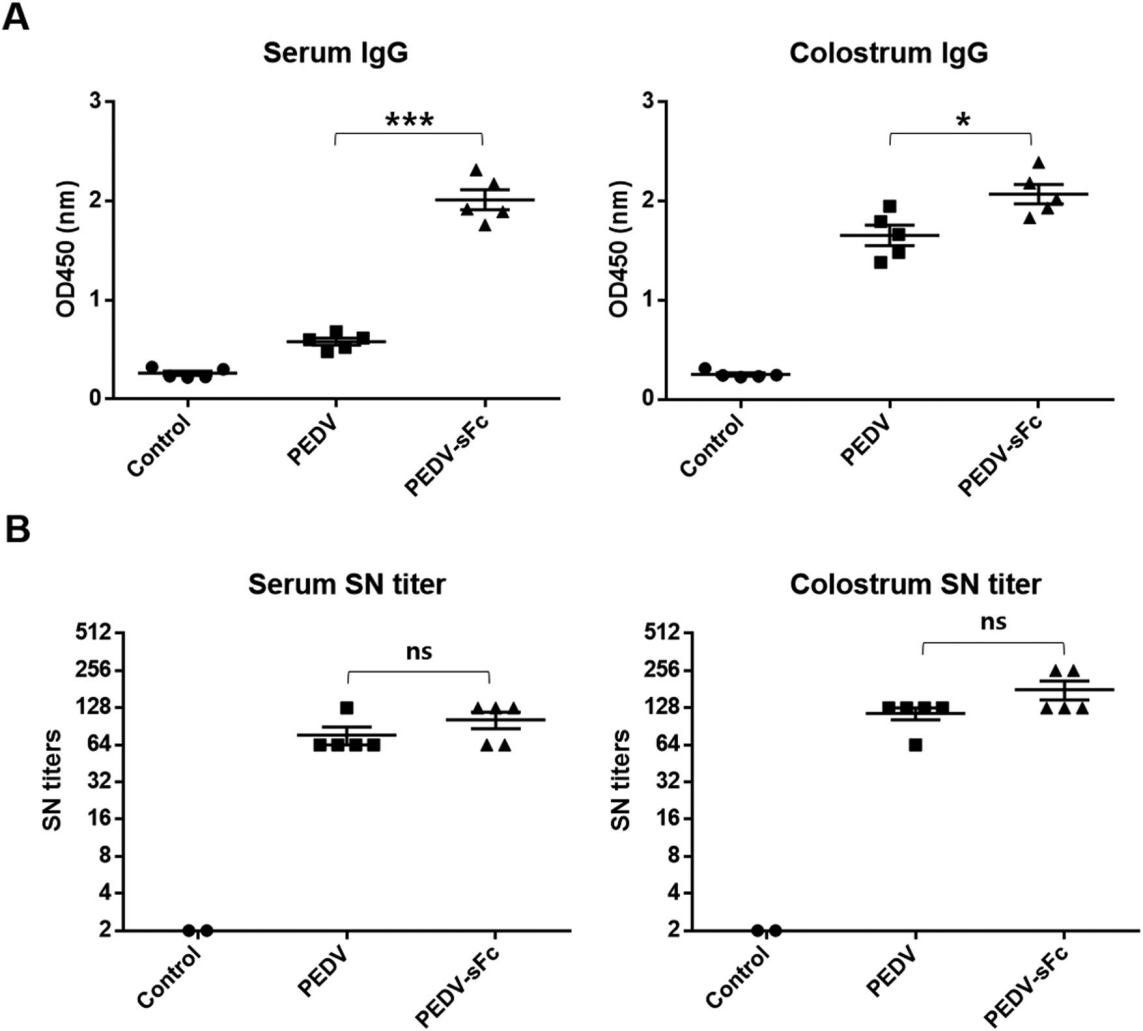
Humoral immune response in pigs immunized with PEDV-sFc. Pigs were immunized with 10^6^ TCID_50_ of inactivated PEDV propagated in Vero or Vero-sFc cells. Serum samples were harvested at farrowing. Colostrum samples were collected on the same day of delivery. (A) The production of IgG against PEDV was detected by ELISA. (B) Serum neutralizing activity was determined by SN test as described in materials and methods. Error bars present SD from the mean. Statistical significance was assessed by student’s t-test. *, *p* < 0.05; ***, *p* < 0.001; ns, not significant.

To determine whether PEDV-sFc could enhance immune response *in vivo*, we vaccinated pigs with PEDV-sFc or PEDV. Serum and colostrum samples were collected from immunized pigs for analysis. First, we determined the titers of PEDV-specific IgG by indirect ELISA. IgG titers in sow sera were significantly higher in PEDV-sFc compared to PEDV (Figure 4A). Similarly, IgG titers in colostrum were also significantly higher in PEDV-sFc than PEDV (Figure 4A).

To further determine the neutralizing activity of antibodies, SN test was performed. The SN titers of serum and colostrum samples were slightly higher than those in pigs immunized with PEDV-sFc, whereas no statistical significance was observed (Figure 4B). Taken together, the results imply that PEDV-sFc immunization induces more effective humoral immune response against PEDV in sow compared to PEDV immunization.

### 3.3. PEDV-sFc stimulates cytokine secretion

**Figure 5.**
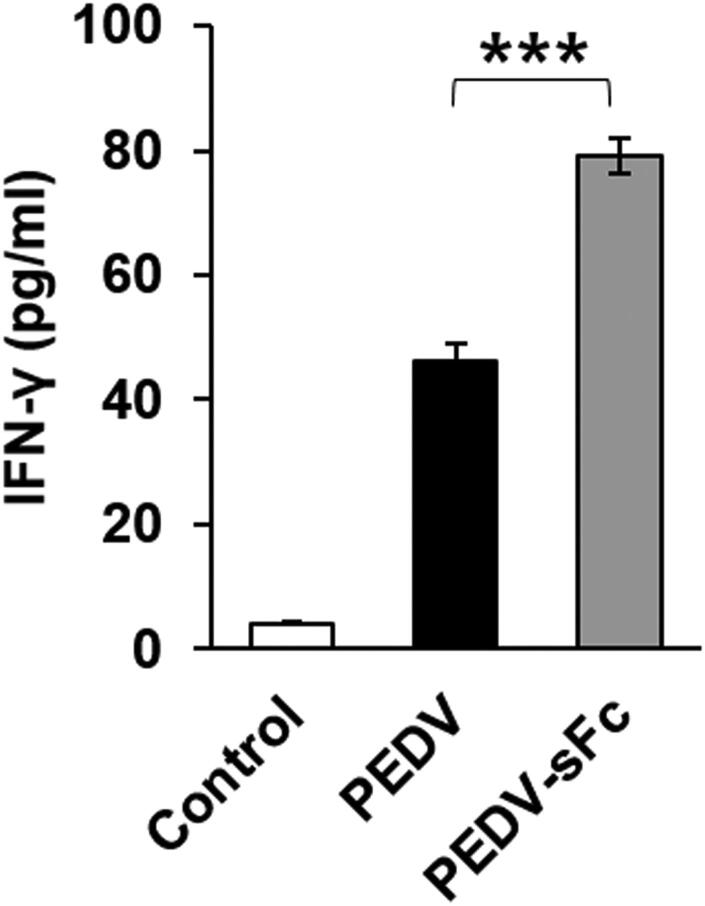
Cytokine secretion against PEDV-sFc. Pigs were immunized with 10^6^ TCID_50_ of inactivated PEDV propagated in Vero or Vero-sFc cells. Serum samples were harvested at farrowing. IFN-γ levels in serum samples were determined by ELISA. Error bars present SD from the mean Statistical significance was assessed by student’s t-test. ***, *p* < 0.001.

IFN-γ is a cytokine that is critical for innate and adaptive immune responses against viral infections. To identify the effect of PEDV-sFc on cytokine secretion, cytokine levels in the serum samples were measured by ELISA. PEDV-sFc immunization showed a significantly increased ability for the induction of IFN-γ secretion compared to PEDV immunization (79 ± 6 pg/ml vs. 46 ± 6 pg/ml, Figure 5). No induction of IFN-γ = was observed in sera from unvaccinated pigs. Taken together, the results demonstrated that the PEDV-sFc elicits the innate and adaptive immunity upon immunization.

## 4. Discussion

In this study, PEDV grown in Vero-Fc cells incorporated host cell derived chimera Fc into the virions (Figure 3). Furthermore, the PEDV-sFc immunized pigs showed the elevated PEDV antibodies and cytokines (Figures 4 and 5).

Antibody Fc region is a recruiter and a frontline commander in the combat against infectious diseases. Therefore, it mediates potent immune effector functions by engaging Fc receptors and serum complement proteins. Fc receptors are crucial mediators of cellular immunity and important link between the IgG secreting lymphocytes and phagocytes (Ravetch and Bolland 2001; Figge 2009). The Fc-Fc receptor interaction heralds the occurrence of virus infection and orchestrates other signaling events that lead to T-cell activation (Amigorena and Bonnerot 1999).

It has been reported that the application of Fc molecules in vaccine development provides opportunities for the enhanced immune responses against various infectious diseases, by engaging Fc receptors found on immune cells. Hepatitis B surface antigen (HBsAg) complexed to human anti-HBs immunoglobulins induced enhanced immune responses (Wen et al. 1999). In addition, a DNA vaccine coding for hepatitis B virus antigen-Fc fusion proteins had enhanced antigen-specific CD4+, CD8+ and B cells (You et al. 2001). The immunization of mice with pseudorabies virus incorporated chimera Fc has induced higher level of IgG, IgG1 and IgG2a and protected mice more efficiently from lethal challenge comparing to the immunization with normal inactivated virus (Takashima et al. 2005). We previously reported that immunization of chicken with avian metapneumovirus MPV (aMPV) harboring chicken Fc induced higher level of antibodies and inflammatory cytokines (IFN-γ and IL-1β) compared to those of aMPV (Paudel et al. 2016).

Besides neutralizing antibodies, the cellular response to PEDV is characterized by T helper cells that are supporting the production of antibodies and cytotoxic T cells that are targeting the infected cells (Meng et al. 2013). In the present study, systemic T cell activity was investigated *in vitro* by the detection of IFN-γ release in serum samples after PEDV vaccination. IFN-γ is produced by T lymphocytes and natural killer cells, and activates macrophages and in this way is involved in both innate and adaptive cell-mediated immune responses (Schoenborn and Wilson 2007). There was significant up-regulation of IFN-γ from PEDV-sFc to that of PEDV (Figure 5). We speculate that the enhanced cytokines facilitated the increased antibody titers in PEDV-sFc immunized groups.

## 5. Conclusions

In conclusion, we showed the ability of PEDV harbouring Fc as an inactivated vaccine. Although inactivated vaccine is safer that live vaccine, inactivated vaccine is generally less effective than live vaccine. The PEDV harbouring Fc has a possibility as an inactivated vaccine, which have comparable effect to live attenuated vaccines. It is worth to compare the ability of the inactivated PEDV harbouring Fc with currently used live attenuated vaccines in pigs. In addition, a challenge experiment would be needed to investigate the protective efficacy of PEDV harbouring Fc.
